# Which is the ideal sanction for cooperation? An experimental study on different types of third‐party sanctions

**DOI:** 10.1002/pchj.259

**Published:** 2018-12-27

**Authors:** Gonglin Hou, Fei Wang, Jieyan Shi, Weijiang Chen, Jie Yu

**Affiliations:** ^1^ Department of Psychological Research Zhejiang Sci‐Tech University Hangzhou China; ^2^ Department of Economics Claremont Graduate University Claremont California USA

**Keywords:** cooperation, public goods, punishment, reward, third‐party sanctions

## Abstract

Cooperation is the crux of many social problems, thus third‐party sanction, as one of the most important ways to promote cooperation, is worth studying. The present study compared the effects of third‐party punishment alone, third‐party reward alone, and the combination of third‐party reward and third‐party punishment on cooperation in the context of a public goods experiment. In addition, we explored the characteristics of third‐party sanctioning behaviors. A total of 280 students participated in the present study. The results showed that the operation of third‐party sanctions did raise the level of cooperation and changed the discrete trend of cooperation—specifically, the differences between the cooperation level of every group member and the average level of the whole group. Furthermore, when third‐party rewards and third‐party punishments were used simultaneously in the public goods game (PGG), the level of cooperation was greatly enhanced, which meant that in the context of the third party, when punishment opportunities and reward opportunities coexist, they may have a “compensatory effect.” In terms of the characteristics of sanctioning behaviors, the frequency of third‐party sanctions did not differ significantly under different conditions (third‐party punishment alone, third‐party reward alone, and a combination of third‐party reward and third‐party punishment), and neither did expenditures on third‐party sanctions, but the strategies that third parties used changed under different conditions, thus their effects on other group members’ cooperative behavior varied under different conditions. The present study provides a comprehensive picture of how third parties behave in different conditions of third‐party sanctions and how these sanctions exert influence on cooperation. Understanding these mechanisms can help us build more effective institutions.

In human society, economic and social activities relating to the provision of public goods require voluntary cooperation. During cooperation, the group faces the public goods dilemma—a conflict between private interests and collective interests (Olson, 1965). Due to human selfishness, some people free ride to get higher personal benefits. If free riding spreads, cooperative social norms will not be followed and scarce public resources will collapse (Dawes & Thaler, 1988; Fehr & Gächter, 2000; Fehr & Schmidt, 1999; Fischbacher & Gächter, 2010; Ledyard, 1994; Sefton, Shupp, & Walker, 2007). Some studies have demonstrated that many people are willing to punish others for violations of social norms or to reward others for good behaviors at personal cost (Fehr & Fischbacher, 2003; Fehr, Fischbacher, & Gächter, 2002), even with their personal costs not repaid (Bowles & Gintis, 2004; Fehr & Fischbacher, 2003; Fehr et al., 2002; Gintis, 2000). Thus, altruistic punishment (negative sanction) and altruistic reward (positive sanction) are considered to be important means to prevent free riding in cooperation (Fehr & Fischbacher, 2004; Fehr & Gächter, 2002; Gürerk, Irlenbusch, & Rockenbach, 2006; Masclet, Noussair, Tucker, & Villeval, 2003; Rand, Dreber, Ellingsen, Fudenberg, & Nowak, 2009; Sigmund, Hauert, & Nowak, 2001; Sutter, Lindner, & Platsch, 2009).

Many studies have tried to explore the effects of punishment and reward on cooperative behavior in the public goods game (PGG)—a classic paradigm that can be used to simulate the real‐world cooperation scenarios in laboratories. It is noteworthy that many previous studies have examined the role of second‐party sanctions in reducing free‐riding behavior.^1^ However, in real life, people may also receive sanctions from third parties—the outcome of whose sanctioning behaviors are unrelated to their own interests. When exploring the effect of third‐party sanctions on maintaining cooperation, most studies have focused on third‐party punishment (TP), but not on third‐party reward (TR) or a combination of third‐party punishment and third‐party reward (TP/R; Lotz, Okimoto, Schlösser, & Fetchenhauer, 2011; Sutter et al., 2009). However, punishment alone cannot increase profits (Bone, Wallace, Bshary, & Raihani, 2015; Dreber, Rand, Fudenberg, & Nowak, 2008), and it even has a negative correlation with profits. Furthermore, punishment is unpleasant; for example, in the case of second‐party sanctions, punishment usually increases the possibilities for revenge (Bone et al., 2015; Janssen & Bushman, 2008). Thus, focusing just on punishment, we cannot determine precisely what people's responses to sanctions (punishments or rewards) are or what sanction enforcers’ responses to norm violations are (Lotz et al., 2011).

Recently, some researchers began to pay attention to the effects of TR alone, TP alone, and TP/R on free riders. Most of them focused on the factors influencing reward and punishment, such as the availabilities of sanctions (Nikiforakis & Mitchell, 2014), the types of social dilemmas (Molenmaker, de Kwaadsteniet, & van Dijk, 2014), and the heterogeneities of groups (Kamijo, 2014); however, the direct impacts of third‐party sanctions on the behavior of the players of the PGGs were ignored. Although some researchers have applied experimental and theoretical models to examine the effects of different types of sanctions (punishments alone, rewards alone, or a combination of rewards and punishments) on cooperation (Andreoni, Harbaugh, & Vesterlund, 2003; Chen, Sasaki, Brännström, & Dieckmann, 2015), their studies have some important limitations. For instance, in Chen et al.’s (2015) research, they used a model to show that the combination of punishments and rewards was the best way to establish cooperation, but they did not conduct any experimental research. Although in Andreoni et al.’s (2003) research they modified the experiment to include the combination of punishment and reward, in their design participants could choose only one type of sanctions (punishment or reward), while in real life, people not only offer the cooperators rewards, but also penalize free riders during the same period of time. Whether or not there is a compensatory effect when people could reward cooperators and penalize free riders during the same period of time has yet to be found.

As an important way to promote cooperation, third‐party sanctions take different forms. The effects of different third‐party sanctions, especially TP/R, have not been explored systematically and how third parties use sanctions to influence cooperation is also unknown, which may limit our understanding of cooperation and weaken our capability to promote cooperation. We note that recently, when investigating cooperation, rather than paying attention almost exclusively to the average level of cooperation, some researchers (Fehr & Gächter, 2000; Sasaki, Brännström, Dieckmann, & Sigmund, 2012; Sefton et al., 2007) have begun to realize the importance of the characteristic of dispersion within cooperation, by paying attention to the question of whether the behaviors of all members concentrated toward cooperation or free riding, for example, or whether the behaviors varied in a more discrete fashion. Besides, they also cared about how far the cooperation levels of individual members deviated from the average cooperation level of the group as a whole. This is because when we talk about cooperation, it would not be complete if we only cared about one characteristic—it is better to investigate cooperation with varied indicators; thus, the discrete trend indicator, which can help us understand the behavior mechanism of different members in a team, is worth studying. In other words, by investigating the deviations from the cooperation levels of group members to the mean contribution level and calculating the frequency of different deviations, we can shape the dispersion curve of group members’ cooperation behaviors and measure to what degree group members behaved far from the average level, which we can regard as the discrete trend of cooperation behaviors. This discrete trend can help us determine whether or not the third‐party sanctions would regulate the group members and lead to a unified rule of cooperation. However, there has been no research yet that has highlighted the discrete trend of cooperation as a special indicator.

Thus, dissecting how different types of third‐party sanctions (third‐party reward, third‐party punishment, and especially the combination of reward and punishment) affect cooperation (both the level of cooperation and the discrete trend of cooperative behaviors) in PGGs is important for exploring cooperation mechanisms. Giving experimental evidence to this question is exactly what we want to do in the present study.

## Hypotheses

Considering that both the level of cooperation and the discrete trend of cooperative behaviors are important characteristics of cooperation, we developed several hypotheses on how TR, TP, and TP/R affect levels of cooperation and discrete trend of cooperative behaviors. Besides, regardless of the conditions, it is sanctions that third parties use to influence cooperation. It is worthwhile paying attention to the sanctioning behaviors of third parties, so we also developed a hypothesis about the sanctioning behaviors of third parties.

Sutter et al. (2009) examined the influence of third‐party intervention on cooperation in a prisoner's dilemma (PD) experiment. They found that third‐party observation and third‐party rewards could improve cooperation levels; however, rewards were given so late that they could not prevent the steady decline of cooperation (Sutter et al., 2009). Since the motivational mechanism for cooperation in PGGs is similar to that in PD games, in practice, a PGG is always viewed as a generalized PD game (Boyd & Richerson, 1992; Camerer & Fehr, 2002; Ledyard, 1994; Levitt & List, 2007). Thus, the effect of sanctions in the context of PGGs may be similar to that in the context of PD games, and the weakness of rewards in the context of PGGs may be similar to that in the context of PD games; therefore, we believe that TP is more useful than TR in facilitating cooperation in PGGs. As to TP/R, Charness, Cobo‐Reyes, and Jiménez (2008) examined the effect of the probability of third‐party intervention on the behavior of the participants in an extension of the investment game. In the context of the investment game, both the investor and responder receive endowments at the beginning, the investor who moves first can decide how many endowments to give to the responder, and then the responder decides how many to give back; thus, in this game, the proportion of endowments that the investor or responder gives out could represent the level of cooperation. Charness et al. found that both in the treatment with punishment and in the treatment with a combination of punishment and reward, the proportion given back by responders and the proportion given by investors were larger than those in the control treatment. Furthermore, the proportion in the treatment with a combination of punishment and reward options was smaller than that in the treatment with punishment opportunities. However, things would be different if the enforcer could use penalties and rewards simultaneously to motivate the cooperation behavior of the same person.^2^ In Andreoni et al.’s (2003) two‐person proposer–responder games, proposers divided a fixed pie and responders enforced punishment/reward to proposers. They found that in the treatment with a combination of punishment and reward options, punishment and reward acted as complements in motivating proposers to increase their offers, which meant that punishment could force the selfish people to change their behavior and to try cooperation; at the same time, the reward could promote further cooperation, rendering the penalty a seldom used but necessary tool (Andreoni et al., 2003). Enlightened by the viewpoint of Andreoni et al., we think the reason behind this may be that penalties and rewards might act as complements in enhancing cooperation in the situation and we argue that this phenomenon would happen in PGGs too. Although in the context of PGGs, the people who receive punishment are not the same people who receive reward, we still believe that a combination of rewards and punishments will lead to higher levels of cooperation than punishment alone considering that the people who receive punishment and the people who receive reward have the same status and that the punishment of non‐cooperative people is another method of rewarding cooperators and vice versa.

From the theoretical perspective, according to Kahneman's prospect theory (Kahneman & Tversky, 1979), the value function commonly used is concave for gains, convex for losses, and flatter for gains than for losses. That is to say, most people demonstrate greater sensitivity to probable losses than to probable gains, and the feelings elicited by the losses are much more intense than the feelings elicited by the gains. In the context of TP, players will be punished and suffer losses if they free ride, so they will prefer to raise the level of cooperation if they want to avoid punishment, while in the context of TR, players will be rewarded and get benefits if they cooperate, therefore the players will prefer to raise the level of cooperation in order to earn rewards. Since compared with probable gains, people are more sensitive to probable losses, punishments will be more effective in raising cooperation levels than rewards. In the context of a TP/R, players will be punished and suffer losses if they free ride, otherwise they cannot only avoid losses, but also get rewards. The contrast and the complementarity between punishment and reward may make players more willing to improve cooperation, thus TP/R will be more effective in improving cooperation than TP alone.

According to the above viewpoints, we developed the following hypothesis:

*Hypothesis 1*: TP, TR, and TP/R all have positive effects on levels of cooperation. In all three treatments, *ceteris paribus*, the level of cooperation will be highest under the TP/R condition, a little lower under the TP condition, and lowest under the TR condition.

When examining the effects of altruistic punishment in motivating cooperation between acquaintances and cooperation between strangers, Fehr and Gächter (2000) discovered the differences in the emergence of common standards relating to individual contributions. They found that in the second‐party punishment condition, if the group composition did not change across periods, a common behavioral standard of cooperation would emerge, but they did not conduct further empirical studies of common standards. In fact, the studies of common standards provided insight on new ways to explore cooperation: Cooperation in a finitely repeated PGG is not only characterized by the mean contribution level of the group members but also by the discrete trend, which usually showed as a player's deviation from the mean contribution level of the other three group members. Pure free riders and conditional cooperators coexist in society (Croson, 2007; Fehr & Gächter, 2000; Fischbacher, Gächter, & Fehr, 2001). Without any sanction, conditional cooperators have no other choice but to defect so that they can avoid being exploited or show their resentment and disappointment when they face defections, and then the aggregate level of cooperation will be very low (Fehr & Fischbacher, 2003). In order to explore whether free riders will choose to cooperate and tend to follow the cooperative norms when there is probability of being rewarded or punished, Sasaki et al. (2012) analyzed the interplay of motivations provided by institutions and the influences of voluntary participation. Although Sasaki et al. did not conduct an experiment to test their model, their model offered strong evidence about the dispersion degrees of cooperation. Through the application of game theory and model analysis, their study indicated that rewards would result in the stable coexistence of free riders and cooperators, and the proportion of cooperators was larger when rewards were higher. In contrast, punishment caused the emergence of alternative stable states. After the competition between cooperators and free riders, one or the other behavior would establish. Cooperation or free‐riding, once established, would be considered as a social behavior norm, that is, as long as most people insisted on one behavior, people would not deviate, so that cooperators and defectors could not coexist for a long time, and people's behavior would show as an aggregate pattern.

From the perspective of prospect theory, people are more sensitive to penalty than to reward; thus, avoiding punishments will be more motivating than seeking rewards. During social cooperation, free riders (the people whose level of cooperation is lower) who are punished can choose to improve cooperation or to take revenge; however, in the context of third‐party sanctions, it is barely possible to take revenge, so free riders are very likely to choose to improve cooperation. Therefore, group members will continuously enhance the level of cooperation, which causes the high aggregate level of social cooperation. As to the reward situation, although players who offer the high level of contribution can get rewards, players who offer the low level of contribution or free ride will not be punished, and they can even gain extra benefits by free riding. Therefore, in this situation, free riders may not adjust their behavior in a more cooperative direction.

Thus, we developed the following hypothesis about the discrete trend of cooperation (which equals the dispersion degrees of cooperation in our research):

*Hypothesis 2*: In the conditions with punishment (TP and TP/R), *ceteris paribus*, the aggregate level of cooperation will be higher and the pattern of contribution behaviors will not be divergent. However, in the TR condition, the pattern of contribution behaviors will be divergent.

Several studies have explored how people behave when they use sanctions. Most of those studies found that people did have preferences when they chose their approach to exert influence on cooperation. Some researchers did experimental social dilemma research to explore this issue in the PGG and found that people would like to use rewards rather than punishments. For instance, Sutter, Haigner, and Kocher (2010) explored which approach people would endogenously choose to facilitate cooperative behaviors. They found that group members typically preferred to reward rather than punish even though punishment was the better way to maintain cooperation. Similarly, Gürerk, Irlenbusch, and Rockenbach (2009) also found that when facing different forms of sanctions, typically, team leaders showed an initial preference for reward rather than for punishment. Specifically speaking, their result showed that 19 out of 20 leaders chose rewards whereas only one leader chose punishments (Gürerk et al., 2009). Lotz et al. (2011) explored people's preference for punitive action and compensatory action, and investigated other emotional determinants and boundary conditions in the context of a modified experimental game. Similar to previous studies, they also found that people chose to compensate cooperators (reward) rather than punish offenders (punish).

Moreover, besides the experimental social dilemmas research on the PGG, there have been other studies conducted to explore people's preferences for different forms of sanctions (punishment and reward). For instance, Baron (1995) explored the decision‐maker's preferences in some hypothetical scenarios, such as the scenario including the decision to divide income between two groups and the scenario including treatment cures for the patients between two groups. Their results showed that when making judgments (decisions), people will apply the “do no harm” principle, which means they are reluctant to reduce some people's benefits in order to help others. According to this viewpoint, in our experimental setting, third parties can reward the cooperators without harming the interests of free riders in treatments where people can use rewards. However, in treatments where people can only use punishments, because of the do no harm principle, the only thing third parties can do is to reduce the punishment.

According to the above literatures and viewpoints, we developed the following hypothesis:

*Hypothesis 3*: When imposing sanctions on people who participate in cooperation programs (PGGs), third parties may prefer to use rewards rather than use punishments. Specifically speaking, under the TR and TP/R conditions, third parties will make more sanctions (rewarding cooperators), while under the TP condition, third parties will make fewer sanctions (punishing free riders).

When we investigated sanctioning behaviors of third parties, it seemed inappropriate to talk about third‐parties’ sanctioning behaviors without the cooperation behaviors because third parties have to conduct sanctions according to the cooperation situation in the PGG; thus, we realized that sanctioning behaviors should be measured, but also that the relationship between sanctioning behaviors and cooperation should be discussed. Previous studies have paid attention to sanctioning behaviors, and among those studies, some investigated the relationship between cooperation and sanctioning behaviors in the context of second‐party sanctions or in the context of another experimental paradigm. Andreoni et al. (2003) examined punishment and reward in a series of two‐person proposer–responder games and they found a subtle and interesting relationship among reward, punishment, and cooperation under varied conditions. For instance, when comparing the condition “carrot” to the condition “carrot–stick,” they found that, for a similar level of cooperation, the average reward would become larger in the carrot condition because of the absence of the ability to punish, which means the relationship between sanctioning behaviors and cooperation is indeed influenced by the conditions. Sefton et al. (2007) examined how sanctions/rewards were used in the context of second‐party sanctions, and found that the determinants of being punished or rewarded varied in the reward condition, the punishment condition, and the reward–punishment condition, which also means that the relationship between sanctioning behaviors and cooperation differed in different sanctioning conditions.

As we have enumerated and hypothesized above, third parties would make different sanctions under different conditions; thus the relationship between third‐party sanctions and cooperation may also vary. According to previous findings in the second‐party context or other similar paradigms, we thus could speculate that in the context of PGG, the relationship between third‐party sanctioning behavior and cooperation would be different according to different sanctioning conditions.

According to this, we developed the following hypothesis:

*Hypothesis 4*: Under different sanctioning conditions, third parties would adjust their strategy of using sanctions according to the conditions, meaning that the relationship between sanctioning behaviors and cooperation would not be the same under different conditions.

## Method

### Participants and treatment conditions

A total of 280 participants—137 males and 143 females—played PGGs in our experiments. All participants were undergraduate or graduate students from all disciplines except economics. Prior to the experiment, all participants had read and signed the informed consent document to express their willingness to participate.

Participants who engaged in the experiment did not know each other and they could not talk with each other during the experiment. Each participant in the experiment was identified by a randomly assigned number and the identification number did not change during the experiment. The participants were not aware of each other's numbers. The decision information and feedback information were transferred by cards. All cards were collected and distributed, face‐down; therefore, in our experiment participants were not completely anonymous, since each participant could be aware of the contributions other group members had made in previous rounds. Scenarios like this mimic real life. In the real world, people do not usually ignore others interacting with them; instead, the behavior towards others is usually influenced by the behaviors of others in the past (Rand et al., 2009).

In order to study the different effects of different types of third‐party sanctions on cooperation, four treatment conditions were set: baseline (B), TP, TR, and TP/R. Each treatment consisted of 10 groups.^3^ In the B treatment, each group consisted of four participants. In the other three treatments, each group consisted of eight participants, half of whom played the PGG (PGG‐player), and the others played the role of third parties (TP‐player).

### Design and procedure

Ethical approval was obtained from the Ethics Committee of Zhejiang Sci‐Tech University. All participants provided written informed consent. Students from Zhejiang Sci‐Tech University joined the experiment voluntarily. They were informed about the scientific procedures—we would collect, analyze, and publish data about their behaviors in a way that the association between their real‐world identities and their behaviors in the games would not be released. There were no additional ethical concerns beyond what is mentioned above. All the participants were anonymous.

In our experiment, we used the standard PGG paradigm as the basic contribution stage in four treatment conditions: In the B treatment, each period only contained a contribution stage; in the other three conditions, each period contained a contribution stage and a decision stage.

The B treatment served as a baseline treatment. In this treatment, there was a contribution stage in each period, in which a group of four PGG‐players ( *i* = 1, 2, 3, 4) played the 10‐period simultaneous‐move PGG. Each group member was given *y* = 20 tokens at the beginning of each round and could voluntarily decide to invest *g*_*i*_ tokens (0 ≤ *g*_*i*_ ≤ *y*) into a common pool and keep 20 − *g*_*i*_ tokens. Total contributions to the common pool G were multiplied by *k* = 1.6 and then distributed equally among the four group members. The earnings that the player obtained each round were *E*_*i*1_, where *i* represents the number of the player and *1* represents the first stage (here the first stage was the contribution stage and there was only a contribution stage in the B treatment). The amount of earnings consisted of two parts: the benefits from the common‐pool resource and the tokens the player kept. Therefore, *E*_*i*1_ = 20 − *g*_*i*_ + 1.6*G*/4. At the end of each round, with the feedback card in hand, every player could become aware of the contributions that other group members had made and their own earnings for this round.

In the TP treatment, TR treatment, and TP/R treatment, each round contained a contribution stage and a decision stage. During the decision stage, there were four participants who simultaneously took the role of third parties in our experiment. However, in order to eliminate the personal bias error, we just randomly selected one of the third parties’ decisions to implement, which meant that each of the four PGG‐players would receive punishment or reward from only one TP‐player. Furthermore, the third parties would not know that only one decision would be executed, and would not be informed whether or not their decisions were implemented. The decision that each third party made would cause a monetary cost for them, whether their decision was implemented or not.

In the TP treatment, after the contribution stage, TP‐player j ( *j* = 1, 2, 3, 4) would punish four PGG‐players ( *i* = 1, 2, 3, 4) according to their contributions in the contribution stage. At the beginning of the decision stage, each TP‐player would get *z* = 32 tokens and a card on which the contribution that decisions PGG‐players made in the contribution stage in this round were recorded. Then, Player j decided to punish PGG‐players according to those contributions by assigning deduction points. The number of deduction points that TP‐Player j assigned to PGG‐Player i was *p*_*ji*_ (*j* was the number of the TP‐player who assigned the deduction points to the PGG‐player whose number was *i*; *p*_*ji*_ ≥ 0). Assigning one deduction point cost the TP‐player one token and at the same time, the TP‐player deducted three tokens from the sanctioned player's payoff (Almenberg, Dreber, Apicella, & Rand, 2010; Charness et al., 2008; Fehr & Fischbacher, 2004; Rand et al., 2009). As described above, we just randomly selected one of the third parties’ decisions to implement. The number of deduction points that the PGG‐player received was *p*_*i*_. Thus, at the end of the decision stage, TP‐Player j earned $E_{j2}=32-\sum_{i=1}^{n}p_{\mathrm{ji}}$ , where *j* is the number of the TP‐player who assigned the deduction points to PGG‐Player i and *2* represents the second stage (here the second stage was the decision stage). It is obvious that the more deduction points TP‐Player j assigned, the less he or she earned, while PGG‐Player i's payoff after two stages of the punishment treatment was given by *E*_*i*2_ = 20 − *g*_*i*_ + 0.4*G* − 3*p*_*i*_, where *i* is the number of the PGG‐player and *2* represents the second stage (here it was the decision stage). At the end of each round, with the feedback card in hand, every PGG‐player could become aware of the contributions that all group members had made to the common pool, the number of deduction points they received, and their own earnings.

In the TR treatment, TP‐Player j ( *j* = 1, 2, 3, 4) would reward four PGG‐players ( *i* = 1, 2, 3, 4) according to their contributions in the contribution stage. At the beginning of the decision stage, each TP‐player would get *z* = 32 tokens, which was the same as the TP‐players in the TP treatment. Then, these TP‐players had the opportunity to reward the PGG‐players according to their contributions by assigning increment points. The number of increment points that the TP‐Player j assigned to the TP‐Player i was *r*_*ji*_ ( *r*_*ji*_ ≥ 0). Assigning one increment point cost TP‐Player j one token. So at the end of the decision stage, TP‐Player j earned $E_{j2}=32-\sum_{i=1}^{n}r_{\mathrm{ji}}$. And, as before, the more increment points that TP‐Player j assigned, the less he or she earned. Meanwhile, if the PGG‐player received one increment point, their earnings would be increased by three tokens. As described above, we just randomly selected one of the third parties’ decisions to implement, so the payoff for PGG‐Player i was *E*_*i*2_ = 20 − *g*_*i*_ + 0.4*G* + 3*r*_*i*_.

In the TP/R treatment, the TP‐players could assign both rewards and punishments. One token spent by the TP‐player increased or reduced the PGG‐player's payoff by three tokens, which was similar to that in the TP and TR treatments. If the number of deduction points was *p*_*ji*_ ( *p*_*ji*_ ≥ 0) and the number of increment points was *r*_*ji*_ ( *r*_*ji*_ ≥ 0), then the payoff for TP‐Player j under the TP/R treatment was: $E_{j2}=32-\sum_{i=1}^{n}p_{\mathrm{ji}}-\sum_{i=1}^{n}r_{\mathrm{ji}}$. The payoff for PGG‐player i was: *E*_*i*2_ = 20 − *g*_*i*_ + 0.4*G* − 3*p*_*i*_ + 3*r*_*i*_.

In the whole experiment, each participant could only participate in one treatment condition. Each participant would spend roughly an hour conducting the experiment. At the end of the experiment, the tokens that participants got during the finitely repeated PGG for 10 periods were exchanged into real money, at an exchange rate of 1 token = ¥0.05 (see Appendix). The PGG‐players earned an average of ¥13.4 ($2.22). The TP‐players earned an average of ¥13.7 ($2.24). The average daily wage of an industrial worker is ¥80 ($12.9) in China. The reward system design is close to the current reality of our country. We used the number of tokens contributed by the PGG‐players in the contribution stage to represent the level of cooperation, used the number of tokens kept by participants after two stages to represent their final payoffs, and used the frequency and number of tokens assigned by TP‐players to represent the frequency of third‐party sanctions and expenditure of third‐party sanctions. Moreover, to investigate the relationship between third‐party sanctions with cooperation, we used the quality (which equaled the PGG‐player's level of cooperation in this study) and the result of cooperation (which equaled the PGG‐player's earnings in this study) to represent cooperation.

## Results

### Changes in level of cooperation under different types of third‐party sanctions

**Figure 1:**
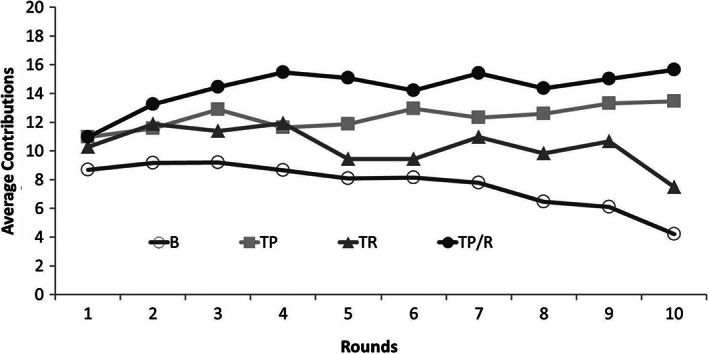
Average contributions for periods 1–10 in four treatments. B = baseline treatment; TP = third‐party punishment treatment; TR = third‐party reward treatment; TP/R = treatment with a combination of third‐party punishment and third‐party reward.

Figure 1 depicts average contributions for periods 1–10 in four treatments. Through the face value of cooperation in every period and trend in Figure 1, we can see that in the baseline treatment without punishments or rewards, the level of cooperation showed a declining trend—cooperation decreased over time and the level of cooperation was rather low in the final period ( *M* ± *SD* = 7.65 ± 3.46, *N* = 10). This result is similar to the findings of many previous studies—it is impossible to maintain cooperation without any threat of sanction (Croson, 2007; Fehr & Gächter, 2002).

In the TP treatment ( *M* ± *SD* = 12.36 ± 2.64, *N* = 10) and the TP/R treatment ( *M* ± *SD* = 14.39 ± 2.02, *N* = 10), the average level of cooperation was kept steadily at quite a high level and the differences between rounds were much smaller than those in the baseline treatment. The Mann–Whitney *U* test showed that the average level of cooperation under the TP/R treatment was significantly higher than that under the B treatment ( *p* < .001), and that the average level of cooperation under the TP treatment was significantly higher than that under the B treatment ( *p* < .01). The average levels of cooperation were compared in the TP treatment and the TP/R treatment, and no significant difference between the two treatments was found ( *p* = .11). However, when it came to the TR treatment, we found that the trend of cooperation was not similar to that under two other treatments (TP and TP/R)—the average cooperation level ( *M* ± *SD* = 10.33 ± 3.37, *N* = 10) under the TR treatment decreased over time. When we checked out Figure 1 and the numerical value, in all three types of sanctions (TP, TR, and TP/R), the average cooperation level under the TR condition was lowest, that is, among all three conditions, TR had the least positive effect on cooperation. The Mann–Whitney *U* test showed that the average level of cooperation in the TR treatment was significantly lower than that in the TP/R treatment ( *p* < .001), but was not significantly different from that in the B treatment ( *p* = .12), nor was the average level of cooperation in the TR treatment significantly different from that in the TP treatment ( *p* = .19), meaning that TR barely had any effect on promoting the level of cooperation. The results of our analysis indicated that both TP and TP/R could significantly raise the average contribution level, and that TR alone had very little influence on cooperation, and its influence is far less than that of TP and TP/R.

### Changes in dispersion degrees of cooperation under different types of third‐party sanctions

**Figure 2:**
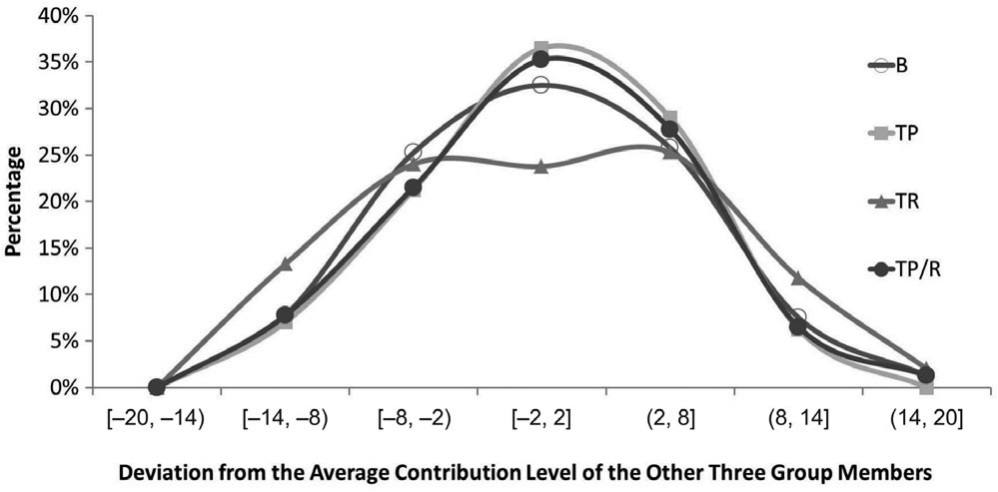
Percentage of observations in the different deviation intervals. B = baseline treatment; TP = third‐party punishment treatment; TR = third‐party reward treatment; TP/R = treatment with a combination of third‐party punishment and third‐party reward.

In order to further explore the effects of different types of third‐party sanctions on contribution behavior, we used an approach similar to those of Fehr and Gächter (2000) and Sefton et al. (2007). When investigating the deviation from the mean contribution level, Fehr and Gächter (2000) and Sefton et al. (2007) firstly calculated the average contribution level of the other three group members, and secondly calculated how far away a PGG‐player's contribution was from average contribution—these are called the deviations. Then they grouped the deviations into seven intervals. In our study, we used the same seven intervals as Fehr and Gächter (2000) used, which are [–20, –14), [–14, –8), [–8, –2), [–2, 2], (2, 8], (8, 14], and (14, 20]. The deviation interval was taken as the abscissa and the frequency of observations in the different deviation intervals was taken as the ordinate. Figure 2 shows that in the B, TP, and TP/R treatments, the PGG‐players’ deviations from the mean contribution level of the other three group members converged on the interval [–2, 2], while the shape of the curve for the TR treatment was quite different from those for the other treatments, which indicated that the contribution behaviors in the TR treatment were more dispersed than those in the B, TP, and TP/R treatments. The chi square test showed that the distribution of the contribution behaviors in the TR treatment was significantly different from that in the B treatment (goodness of fit test, χ^2^ = 36.76, *df* = 5, *p* < .001). From the above, we know that third‐party sanctions would not only influence the level of cooperation, but also change the distribution of cooperative behaviors. When we used “the sum of the absolute deviations from the mean” to represent the “dispersion of cooperative behaviors,” the rank sum test of the dispersion of cooperative behaviors showed that the TP treatment differed significantly from the TR treatment ( *p* = .02). The dispersion degree of cooperative behaviors in the TP/R treatment was between that in the TP treatment and that in the TR treatment, and there was no significant difference between the dispersion degree of cooperation in the TP/R treatment and that in the other two treatments.

The above results indicated that, among the dispersion degrees of cooperation in three treatments (TR, TP/R, and TP), the dispersion degree of cooperation in the TR treatment was the highest, the dispersion degree of cooperation in the TP/R treatment was between that in the TP treatment and that in the TR treatment, and the dispersion degrees of cooperation in the TP treatment were the lowest.

### Characteristics of third‐party sanctioning behaviors

#### *Frequency of use of third‐party sanctions and third parties’ expenditures on sanctions*

In the context of second‐party sanctions, Oliver (1980) has pointed out that due to some details, such as cost types and environment, rewards and penalties might be extremely different for the person giving them. In order to examine Oliver's viewpoint in the context of third‐party sanctions, here we explored the characteristics of third‐party sanctions behavior. The results of the Mann–Whitney *U* test were not consistent with Oliver's claim. The results showed that there was no statistically significant difference in the frequency between positive and negative sanctions (punishment in the TP treatment vs. reward in the TR treatment, *N*_1_ = 10, *N*_2_ = 10, *Z* =  −1.63, *p* = .11; punishment vs. reward in the TP/R treatment, *N*_1_ = 10, *N*_2_ = 10, *Z* =  − 1.33, *p* = .19) and no significant difference between positive and negative sanctioning expenditures (punishment in the TP treatment vs. reward in the TR treatment, *N*_1_ = 10, *N*_2_ = 10, *Z* =  −1.66, *p* = .11, punishment vs. reward in the TP/R treatment, *N*_1_ = 10, *N*_2_ = 10, *Z* =  − 0.34, *p* = .74). The results also show that there was no significant difference in the frequency of sanctions between different types of third‐party sanctions and no significant difference in expenditure on sanctions between different types of third‐party sanctions (frequency of sanctions: punishment in the TP treatment vs. punishment and reward in the TP/R treatment, *N*_1_ = 10, *N*_2_ = 10, *Z* =  −1.89, *p* = .06, reward in the TR treatment vs. punishment and reward in the TP/R treatment, *N*_1_ = 10, *N*_2_ = 10, *Z* =  −0.34, *p* = .74; expenditures on sanctions: punishment in the TP treatment vs. punishment and reward in the TP/R treatment, *N*_1_ = 10, *N*_2_ = 10, *Z* =  − 0.79, *p* = .44, reward in the TR treatment vs. punishment and reward in the TP/R treatment, *N*_1_ = 10, *N*_2_ = 10, *Z* =  −1.29, *p* = .22). That is to say, third parties did not spend more on the combination of reward and punishment in the TP/R treatment than on punishment in the TP treatment (or reward in the TR treatment).

#### *Relationship between third‐party sanctioning behavior and PGG‐player's level of cooperation*

**Figure 3:**
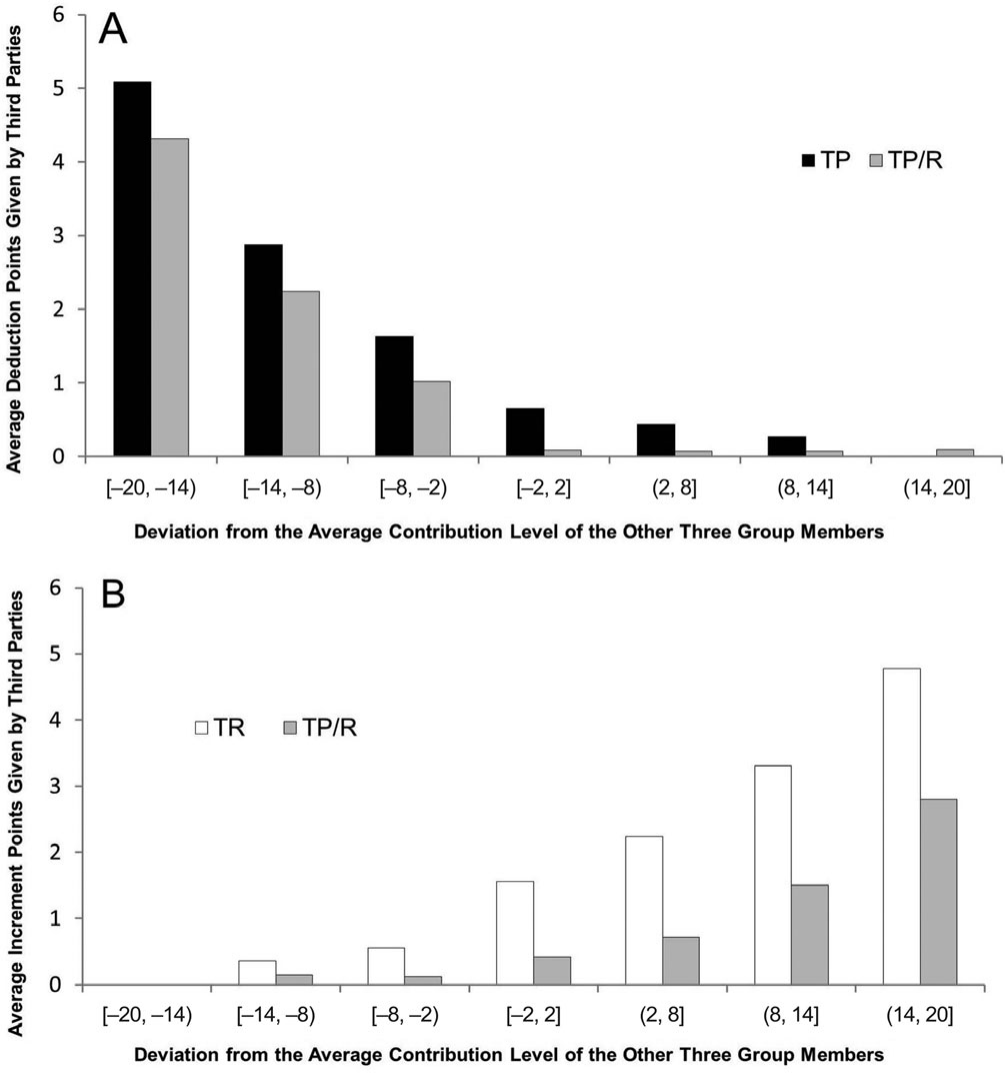
Average (A) deduction points and (B) increment points given by third parties in the third‐party punishment (TP) treatment, third‐party reward (TR) treatment, and treatment with a combination of third‐party punishment and third‐party reward (TP/R).

Knowing the frequency of sanctions and expenditures on sanctions is not enough, and investigating the relationship between third‐party sanctions and cooperation under different conditions may lead to a deeper comprehension of how third‐party sanctions work, so we further analyzed the data. First, we tried to identify the relationship between third‐party sanctions and cooperation by looking at the graph. We calculated the PGG‐player's deviations from the average contribution level and grouped them into seven intervals as we had done before. The deviation interval was taken as the abscissa and the number of deduction points or increment points assigned by a TP‐player was taken as the ordinate. Figure 3A shows that, both in the TP treatment and in the TP/R treatment, the larger the absolute value of the negative deviation from the mean, the more deduction points assigned by third parties were. It is noteworthy that when the cooperation level of a PGG‐player was higher than the average cooperation level of the other group members, some third parties still imposed punishment, but the higher the positive deviation, the less the deduction points assigned by third parties were. Figure 3B shows that, both in the TR treatment and in the TP/R treatment, the larger the absolute value of the positive deviation from the mean, the more the increment points assigned by third parties were. Similarly, when the cooperation level of a PGG‐player was lower than the average cooperation level of the other group members, some third parties still gave rewards, but the higher the absolute negative deviation, the less the increment points assigned by third parties were.

Next we wanted to identify some statistical evidence on the relationship between third‐party sanctions and cooperation, that is, we aimed to characterize the determinants of punishments and rewards through data analysis. Thus, we used an ordinary least squares (OLS) regression model, which is similar to models used by Fehr and Gächter (2000) and Sefton et al. (2007), to explore the relationship between third‐party sanctions and the PGG‐players’ levels of cooperation in the TP, TR, and TP/R treatments. The dependent variable was “average sanction points” assigned to a given PGG‐player, and the independent variables comprised “average contribution of the group,” “positive deviation,” and “the absolute value of negative deviation,” respectively. We calculated the absolute value of negative deviation by taking the absolute value of the negative deviation of a participant's contribution from the average contribution level of the other three players where their own contribution was below the mean. This variable was equal to zero if their contribution was no less than the others’ average contribution. The positive deviation variable was defined analogously. Unlike the experiments by Fehr and Gächter (2000) and Sefton et al. (2007), in which an explanatory variable was “the others’ average allocation,” we used the “average contribution of the group” instead, which was the average contribution of a group of four members. This is because in the research by Fehr and Gächter (2000) and Sefton et al. (2007), the sanction was carried out by one of the PGG‐players and received by the other three members, but in our experiment, the sanction was carried out by TP‐players and received by four PGG‐players. Furthermore, to explain the time effects, we introduced period dummies in the regression. The model also included group dummies to control for fixed effects (Königstein, 2000). Table 1 contains the model and shows the results of OLS regressions separately for the TP, TR, and TP/R treatments.

**Table 1:** Determinants of Third‐Party Sanctions: Regression Results (*N* = 400)

Independent variables	Dependent variable: Average sanction points assigned to a PGG‐player			
	Deduction points in TP treatment	Increment points in TR treatment	Deduction points in TP/R treatment	Increment points in TP/R treatment
Constant	0.798***	0.126***	0.057	–0.189
	(0.187)	(0.266)	(0.127)	(0.157)
Average contribution	–0.017	0.012	–0.001	0.025**
	(0.014)	(0.023)	(0.008)	(0.010)
Positive deviation	–0.040**	0.203***	0.005	0.142***
	(0.014)	(0.020)	(0.007)	(0.009)
Absolute negative deviation	0.224***	–0.079***	0.188***	0.002
	(0.012)	(0.019)	(0.006)	(0.008)
*F*	16.768***	11.009***	30.255***	9.041***
Adj. *R*_1_^2^	0.668	0.561	0.789	0.507
Durbin–Watson	1.925	1.443	1.792	1.405

*Note*. PGG‐player = participant who played public goods game; TP = third‐party punishment; TR = third‐party reward; TP/R = combination of third‐party punishment and third‐party reward. Standard errors in parentheses. These models include period dummies and group dummies to control for fixed effects.

** *p* < .01.

*** *p* < .001.

The results of the regression analyses showed that consistent with the above description, in the TP treatment, the absolute negative deviation had a positive effect on the number of deduction points, the positive deviation had a negative effect on the number of deduction points, and the average contribution of four PGG‐group members had no significant effect on the number of deduction points; in the TR treatment, the positive deviation had a positive effect on the number of increment points, the absolute negative deviation had a negative effect on the number of increment points, and the average contribution of four PGG‐group members had no significant effect on the number of increment points; in the TP/R treatment in which both positive and negative sanctions were available, the number of deduction points was only positively affected by the absolute negative deviation but the number of increment points was affected both by the positive deviation and the average contribution of four PGG‐group members. Moreover, “the third party's probability of assigning sanction points to three of four PGG‐group members in a period” was about 24.5% in the TP treatment, 36.5% in the TR treatment, but only 3.5% in the TP/R treatment. The reasons for this phenomenon may lie in that in the TP or TR treatment, third parties were inclined to replace the combination of penalties and rewards with gradient punishment or reward. This trend is also reflected in Figure 3. In the TP or TR treatment, the number of deduction points or increment points would decrease or increase when the deviation increased from negative to positive, while in the TP/R treatment this phenomenon did not exist. This might be because in the condition under which both punishment and reward were available, third parties did not need to implement non‐reward to substitute for punishment or vice versa; on the contrary, they used a combination of punishments and rewards.

Furthermore, it is also interesting that only in the TP/R treatment, the average level of cooperation of four PGG‐group members had a positive effect on reward decisions made by third parties—in the other treatments, the average contribution level did not have a significant effect on reward decisions made by third parties. That is to say, in the TP/R treatment, four PGG‐group members could get more rewards if the average level of cooperation increased. It is thus clear that, when third parties could use rewards and punishments at the same time, they not only motivated the targeted member by rewards, but also used rewards as a tool to encourage the whole group to pursue bigger collective interests. Overall, when third parties could use punishment alone or reward alone, they were inclined to implement gradient punishment or reward. When they could use both incentives (punishment and reward), they implemented reward and punishment at the same time, moreover, they would use reward as a tool to encourage the whole group to pursue bigger collective interests.

#### *Relationship between third‐party sanction behaviors and PGG‐player's earnings*

**Figure 4:**
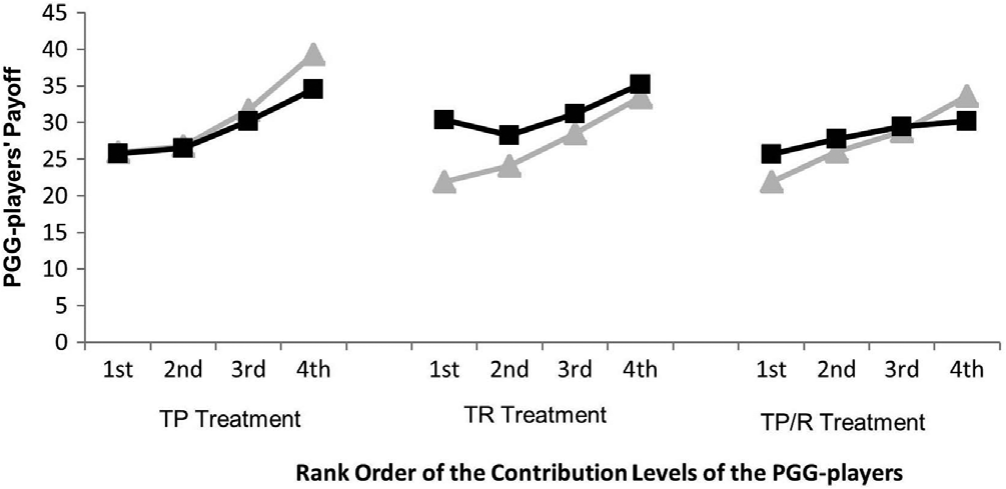
The income of each participant who played the public goods game (PGG‐player) before and after the third‐party intervention. Third‐party sanctions can change the eventual payoffs of PGG‐players in the baseline (B) treatment, third‐party punishment (TP) treatment, third‐party reward (TR) treatment, and treatment with a combination of third‐party punishment and third‐party reward (TP/R). Filled triangles represent PGG‐players’ actual payoffs obtained in the contribution stage, and filled squares represent their presumptive gains after the third‐party intervention.

Obviously, third parties’ behaviors would impact the eventual earnings of the PGG‐players. We ranked the contribution levels of four PGG‐players in each period from highest to lowest, assigning a *1* to the highest, a *2* to the second highest, and so on. In Figure 4, rank order of the contribution levels of the PGG‐players was taken as the abscissa and the income of the PGG‐players was taken as the ordinate. The filled triangles represented the income *E*_1_ that the PGG‐players got in the contribution stage. The filled squares represented the presumptive incomes of the PGG‐players after the third‐party interventions. Because punishment and reward both had a 3:1 technology in the experiment, the earnings that the PGG‐player lost (or gained) = 3× the average deduction (or increment) points assigned to the PGG‐player by the third party in a period. Thus, the filled squares represented $E_{1}-3{\bar{p}}_{i}$ in the TP treatment, $E_{1}+3{\bar{r}}_{i}$ in the TR treatment, and $E_{1}-3{\bar{p}}_{i}+3{\bar{r}}_{i}$ in the TP/R treatment. From Figure 4 we can see that in the punishment treatment, the PGG‐player who contributed most to the common pool got the lowest earnings in the group, the player who contributed least got the highest earnings, and so on. Interestingly, in the TP and TP/R treatments, after the third‐party interventions, the PGG‐players’ earnings were changed because of those deduction points and increment points, but the ranks of their earnings did not change. However, in the TR treatment, the third‐party interventions could change not only the PGG‐players’ earnings but also the ranks of their earnings. After the third‐party interventions, the PGG‐player who contributed most but got the lowest earnings in the contribution stage no longer got the lowest earnings in the decision stage. He or she surpassed the eventual payoff of the player who got the third highest income in the contribution stage, and got almost the same income as the player who got the second highest income in the contribution stage. Such a mechanism changed the embarrassing situation where the participant who contributed most got the least payoff in the contribution stage and made sure that the most cooperative person would not get the lowest eventual payoff in a group.

Overall, the above results showed that TP and TP/R could not change the ranks of the PGG‐players’ earnings, but TR could do so. Third parties tended to offer rather substantial rewards to the PGG‐player who contributed most to the common pool but got the lowest earnings in the contribution stage among the four group members, thus the PGG‐player no longer received the lowest eventual earnings in the decision stage.

## Discussion

Systematically exploring the effects of TP alone, TR alone, and TP/R on social cooperation, our results provide a deeper explanation of the relationship between third‐party sanctions and cooperation under different conditions.

In terms of the level of cooperation, our results show that, no matter which type of third‐party sanction is used, third‐party sanctions have a positive influence on social cooperation. In particular, TP and TP/R had more positive effects on raising the levels of cooperation than did TR, and TR had very little effect on cooperation. This result is similar to findings from many previous experiments in various contexts (Andreoni et al., 2003; Gächter, 2012; Sasaki et al., 2012). Meanwhile, our results are in line with loss aversion under prospect theory (Kahneman & Tversky, 1979), which suggests that people tend to prefer to avoid losses rather than acquire equivalent gains, that is, people are more sensitive to the probable losses caused by punishment than to the probable gains caused by reward, so that punishment is more effective at promoting cooperation than reward. We also found that in the B and TR treatments, cooperation decreased over time, and PGG‐players reached a very low level of cooperation in the last period; and that in the TP and TP/R treatments, the cooperation levels increased over time. The cause may be that under the B condition, in the finitely repeated game, the non‐cooperation of other group members in the next period is a kind of second‐party sanction to the non‐cooperative group member; however, in the final period, the other group members have no chance to choose to free ride in the next round, and then the second‐party sanction does not exist any longer and most of the PGG‐players tend to free ride. Under the TR condition, in the final period, the second‐party sanction does not exist any longer and, without punishment, the impact of rewards from third parties is limited; therefore, most of the PGG‐players tend to free ride in the last round. Under the TP and TP/R conditions, the second‐party sanction does not exist in the final period, but punishments from third parties have a positive impact on cooperation.^4^

Our results show that the TP and TP/R significantly raised the average level of cooperation. In particular, though there was no statistically significant difference between TP and TP/R, when we check Figure 1 and check the numerical value of average contribution, we can see that TP/R led to quite a high level of cooperation among all three conditions. This potential tendency of improving cooperation is worth noting. Actually, this result is very similar to the result from Andreoni et al. (2003)’s research, which used proposer–responder games, and also found that the responders (second parties) did have demands for both punishment and reward; furthermore, when a combination of punishment and reward was available, the cooperation would be more greatly enhanced than punishment alone or reward alone. In their study, they suggested that second‐party punishment and second‐party reward acted to complement one another. In the current study, we also argued that third‐party rewards and third‐party punishments might act as complements in motivating participants to increase their contributions. Considering the context of third‐party sanctions, we inferred that the complementary effect may be due to the specific sanction strategy used by third parties when they had the option of punishment in combination with reward. With the option of punishment only or reward only, third parties can use only one single form of sanctions, which means that the PGG‐players are influenced by only one form of sanctions. However, with the option of punishment in combination with reward, third parties can use two forms of sanctions simultaneously, which means that the PGG‐players are influenced by two forms of sanctions at the same time. For example, during the sanctioning process, being punished due to Player 1’s free‐riding behavior could exert influence on Player 1’s earning; however, reward for other players due to their high level of cooperation could also change the relative rank of Player 1’s earning, so the contrast between punishment for oneself and reward for other players may also make PGG‐players more willing to improve cooperation and vice versa. This may result in TP/R becoming more effective in improving cooperation than TP alone or TR alone.

In terms of the dispersion degrees of cooperation, Oliver (1980) suggested that there were structural differences between the impact of positive and negative incentives on cooperation behaviors. Oliver believed that positive selective incentive was an effective tool to inspire small numbers of cooperators and created pressures toward smaller, more “elite” behaviors; and that negative selective incentives were efficient for motivating unanimous cooperation. In our experiment, we found that the dispersion degree in the TR treatment was the highest, the dispersion degree in the TP/R treatment was between that in the TP treatment and that in the TR treatment, and the dispersion degree of cooperation in the TP treatment was the lowest. The result that the dispersion degree of cooperation in the TR condition is greater than that in the TP condition supports Oliver's viewpoint. When we viewed the dispersion degree of cooperation as an indictor of the uniformity of cooperation behavior, this result showed that the existence of third‐party punishment led the group members to common cooperation rules—all members turned to a similar cooperation level and thus the differences between group members were much less than in the reward condition. This result may be strong evidence that different types of third‐party sanctions may bring about varied cooperation mechanisms for groups and varied behavior mechanisms for members; specifically, punishment might lead to convergent cooperation rules in a group and reward might lead to decentralized rules. There are two possible explanations for why varied sanctioning conditions lead to different dispersion degrees of cooperation: One is that most people show greater sensitivity to probable losses than to probable gains, and the negative feeling caused by the threat of punishment urges free riders to change their strategy to cooperate and motivates other members to continue to cooperate, while the happiness caused by reward only has an effect on several group members who contribute much and get reward but cannot promote the incentive of the free riders who contribute little and could not get reward for cooperation. The other is that third parties tend to offer rather substantial rewards to the conditional cooperator who contributes most but gains least under the TR condition. Since the third parties’ concerns about contributors can change the unfavorable ranks of conditional cooperators, rewards from third parties can encourage cooperators to sustain their high level of cooperative behavior. However, under this mechanism, the ranks of the earnings of PGG‐players whose contribution levels are near the median will be the lowest. If they want to change the situation, they must change their original contribution strategy to learn from group members who contribute most and increase the level of contribution or to learn from the free riders and contribute less. Thus, the reason why the dispersion degree of cooperation in the reward condition was greater than that in the other conditions might be that in the TR condition, some group members whose contribution was near the median might change their contribution strategy to contribute more or to contribute less, and then the diversity increased.

In terms of third‐party sanctioning behavior, we found that there was no significant difference in the implementation of sanctions in general between different forms of third‐party sanctions—this result is different from Hypothesis 3. The reason may be that some previous studies supporting Hypothesis 3 just investigated the preference rather than the actual behaviors, but in the current study, we investigated the behaviors of people when they used sanctions in the context of PGG; the difference in method and context between our study and previous studies may lead to some differences in results. Furthermore, even in the previous study, which found that people preferred to use rewards rather than use punishments, researchers also found that this preference changed during the experiment: Gürerk et al. (2009) found people who initially chose to use rewards would switch to using punishments when they realized reward was not an effective way to maintain cooperation. Besides, in the situation that only one type of sanctions can be chosen to regulate the cooperation, the usage of sanctions would not be the same all the time. For instance, Rand et al. (2009) found that, in the context of second‐party sanctions, the usage of punishment and reward changed over time; specifically, punishment decreases and reward increases over time. Similarly, Andreoni et al. (2003) and Sefton et al. (2007) both found that the usage of punishment and reward changed as the decision game carried on. Considering that third parties might switch the type of sanctions in the whole process under the TP/R condition or their usage of punishment or reward might decrease or increase over time under the TP and TR condition, when we calculated the “average frequency” of use of punishments and rewards or the “average total expenditure” for punishments and rewards, we found there was no significant difference between different treatments.

Another finding of the current study is about the PGG‐group members’ earnings. Obviously, besides the level of cooperation, the result of cooperation (the participant's earning is usually treated as the result of cooperation) is also an important criterion for evaluating whether cooperation is successful. In our study, the PGG‐group members’ eventual earnings were affected not only by the level of cooperation, but also by the third‐party sanction behaviors. Though there was no significant difference in the implementation of sanctions in general between different forms of third‐party sanctions, there were some differences in details. In the TP treatment or TR treatment, third‐party sanction behavior was influenced only by a PGG‐player's deviation from average contribution; third parties regarded punishment or reward as a tool to eliminate unfairness and they were inclined to use gradient punishment or reward. In the TP/R treatment, third‐party sanction behavior was influenced not only by a PGG‐player's deviation from average contribution, but also by the average contribution of the group, because third parties used reward and punishment jointly to eliminate unfairness in income and they used reward as a tool to improve total social welfare. Those different mechanisms did make differences in PGG‐group members’ earnings. In the TP and TP/R treatments, after the third‐party interventions, the PGG‐players’ earnings were changed, but the ranks of their earnings did not change; however, in the TR treatment, the third‐party interventions could change not only the PGG‐players’ earnings but also the ranks of their earnings. After the third‐party interventions, the PGG‐player who contributed most but got the lowest earnings in the contribution stage no longer got the lowest earnings in the decision stage. He or she surpassed the eventual payoff of the player who originally got the third highest income in the contribution stage and got almost the same income as the player who originally got the second highest income in the contribution stage. Such a mechanism changed the embarrassing situation where the participant who contributed most got the least amount of payoff in the contribution stage and made sure that the most cooperative person would not get the lowest eventual payoff in a group. Kohler (2011) extended a model of inequality aversion (Fehr & Schmidt, 1999) and altruism and used lots of experimental evidence, for instance, the experiment by Andreoni and Miller (2002). Kohler (2011) considered that humans’ pro‐social behavior was not only due to the aversion of inequality, but also linked with the interest in social welfare. Although there are differences in the availability of sanctions between our experiment and Kohler's model, and the third‐party sanction behaviors and their effects on participants’ earnings are different accordingly, our results support the model proposed by Kohler, which indicated that because the cooperation or non‐cooperation of group members in the PGG led to third parties’ inequality aversion and inspired their concerns for the total social welfare, the cooperation or non‐cooperation of group members caused third parties to implement sanctions.

The current study sheds light on the investigation of cooperation and third‐party sanctions, particularly the combination of rewards and punishments. First, by studying the characteristics of cooperation, we were able to provide evidence that third‐party reward and third‐party punishment had different levels of effectiveness in raising the level of cooperation and changing the dispersion degree of cooperation. Particularly, the combination of punishment and reward was influential in promoting cooperation. Second, by exploring third‐party sanctioning behavior, our results provide a deeper explanation of the relationship between cooperation and third‐party sanctions. What is more important is that we found that although there was no difference in the frequency or expenditures of sanctions, differences in strategies that third parties used when intervening with cooperation would lead to different eventual results of cooperation. This reminds us that when conducting future research, researchers should not only care about the superficial characteristics of behaviors, but also pay attention to the differences in details and preferences underlying behaviors.

Finally, there are still some limitations in the current study. First, in this study, we used prospect theory to help us justify the hypothesis of the current study, as many other studies have done; however, some researchers have argued that prospect theory is an individual decision theory that may not always apply in interdependent situations, such as the cooperative context of coalition games and trust games, so whether the results could be established under other paradigms still needs further exploration. Moreover, although we have already tested which aspect of cooperation was affected by third‐party sanctions and how different types of third‐party sanctions affected cooperation from the perspective on behavioral economics, the mechanisms of evolution of cooperative behaviors (e.g., the evolution of human reciprocity) from a psychological perspective and from a sociological perspective are still under investigation. For example, we investigated the dispersion trend of cooperation as an indictor to judge the uniformity of cooperation rules. This investigation of the dispersion trend of cooperation offered a new perspective, which may help us to note not only the average level of cooperation, but also the behavior mechanism of cooperation. However, will this dispersion trend represent the cohesion of group members? And will this dispersion trend show or exert influences on cooperation psychology? There are lots of questions that still need to be investigated. Furthermore, two fundamental questions still remain unanswered: (1) What is the motivation of third‐party behaviors? and (2) What is the mechanism behind the relationship between sanctioning behaviors and cooperation?

## Conclusions

Based on the above results and discussion, we have drawn several conclusions. First, third parties were willing to punish free riders or reward cooperators at personal cost to maintain social cooperation, and these sanctions indeed promoted cooperation. Specifically speaking, reward alone barely had any effect on cooperation; punishment alone had significant effects; and the combination of punishment and reward showed a huge advantage—at least a huge potential advantage—on affecting cooperation, which may be due to the “complementary effect.” Second, third‐party sanctions affected not only the level of cooperation, but also the dispersion degree of cooperation—reward alone from a third party led to divergence of contribution behavior, and the other two types of sanctions led to convergence of contribution behavior. Third, during the sanctioning process, the frequency of third‐party sanctions and expenditures on sanctions did not vary under different conditions, but the strategies that the third party used were different under different conditions, meaning that third parties continuously adjusted their sanctioning behaviors according to the tools available. As a result, differences in third parties’ decision‐making procedures and differences in strategies that a third party uses under different conditions led to different eventual payoffs for cooperation.

## Disclosure of conflict of interest

The authors declare that there are no conflicts of interest.

## Notes

1 Second‐party sanctions are sanctions enforced by people whose own interests will be affected by the outcome of the cooperative behavior. For instance, second‐party sanctions can be sanctions from the group member whose own interests will be affected by his or her own cooperative or uncooperative behaviors and other group members’ cooperative or uncooperative behaviors.

2 In Charness et al.’s (2008) study, the enforcers used penalties to motivate the cooperation behavior of the respondents and used rewards to motivate the cooperation behavior of the investors.

3 According to the characteristics of the data, we planned to use non‐parametric test methods when conducting statistical tests. For the non‐parametric test, which is not so strict in regards to sample size, 10 groups for each treatment is enough. Furthermore, we referred to many previous studies and found that many (such as Fehr & Gächter, 2000; Sefton et al., 2007; Sutter et al., 2010), especially those in the context of the PGG, set the sample size as 10 groups—or even fewer—per condition; thus we set our sample size as 10 groups.

4 Implementing backward induction in the finitely repeated game, which has only two players who have the choice of two strategies, we find that the Nash equilibrium is mutual free riding. However, a game can have two players who have the choice of three strategies, which may result in multiple Nash equilibriums. In that case, we can find the Nash equilibrium in the first stage may be cooperation. In the baseline treatment in our experiment, there were four PGG‐players, each of whom had a choice of 21 strategies in each period, so multiple Nash equilibriums might exist in this game. Furthermore, the PGG‐players who did not learn game theory before could not compute Nash equilibrium within the limited time. Thus, during the first period to the ninth period, the PGG‐players who followed the win–win philosophy chose to contribute different amounts, and the rest of the players chose to free ride. If some PGG‐players chose to free ride in this round, there were more people who chose to free ride in the next round, which was a kind of second‐party sanction, so the cooperation level decreased over time. Also, the PGG‐players knew that there was no second‐party sanction in the final round, therefore most PGG‐players chose to free ride in the final period.

## 1. Instructions to the PGG‐players to participate in the investment game in the TP/R treatment

This is an experiment in decision‐making. You may earn tokens in the experiment. The number of tokens you acquire depends on your decisions in the experiment. When the experiment is over, the tokens you obtain can be exchanged into money, at an exchange rate of 1 token = ¥0.05. It is prohibited to talk with the other players during the experiment.

You and the other three group members will play the investment game together. Each member of your group is randomly assigned a number and is named *Player[number]*. The identification number does not change during the experiment. There is another participant, a TP‐player, who will not play the investment game, but participate in the experiment as the supervisor of your group. **This experiment consists of 10 rounds, and each round of the experiment has two stages. The group composition remains constant across periods.**

### Stage 1

In Stage 1 of each round, you will play the investment game and each of the four members of your group is endowed with 20 tokens. You have to determine the number of tokens to contribute to the project and the remaining tokens are yours to keep. Contributions made by all four players to the group project are multiplied by 1.6 and then distributed equally among all four players. Thus, **your income at Stage 1** is: **20 – (your contribution to the group project) + 1.6 × (contributions made by all four group members)/4**. Here are two examples:

Example 1: Each member invests 20 tokens to the project, then each member receives 20 − 20 + 1.6 × (20 + 20 + 20 + 20)/4 = 32 tokens.

Example 2: Three members contribute all 20 tokens and one member contributes zero tokens, then each contributing member receives 20 − 20 + 1.6 × (20 + 20 + 20)/4 = 24 tokens, and each non‐contributing member receives 20 − 0 + 1.6 × (20 + 20 + 20)/4 = 44 tokens.

### Stage 2

In Stage 2 of each round, the TP‐player will participate in the experiment as the supervisor of your group. He or she is endowed with 32 tokens in each round. At the beginning of the stage, he or she is informed about the contribution that each player has made in the investment game and then he or she can reduce or increase the payoff that each player receives in the investment game by assigning deduction points (P) or increment points (R). Assigning 1 deduction point costs the TP‐player 1 token and at the same time, he or she deducts 3 tokens from the sanctioned player's payoff; assigning 1 increment point costs the TP‐player 1 token and augments the payoff of the rewarded player by 3 tokens. For example, if the TP‐player assigns x deduction points to you, he or she will lose x tokens and you will lose 3x tokens; if the TP‐player assigns y increment points to you, he or she will lose y tokens and you will receive 3y tokens. The TP‐player will decide how many deduction points or increment points he or she wishes to assign to each player who participates in the investment game. Thus, **your net earnings for this round will be calculated as follows**: **(your gross earnings from Stage 1) – 3 × (the number of deduction points you get) + 3 × (the number of increment points you get)**.

At the end of each round, with the feedback card in hand, you can become aware of the contributions that the other group members have made to the group project, the number of deduction points and increment points the other group members have obtained, and your earnings for this round. After knowing the results of this round, you will go to the next round. Each round of the experiment has the same stages. The net earnings that you make each round will be totaled and paid to you in cash after the experiment is over.

## 2. Instructions for the TP‐players in the TP/R treatment

This is an experiment in decision‐making. You will receive tokens in the experiment. When the experiment is over, the tokens you get can be exchanged into money, at an exchange rate of 1 token = ¥0.05. It is prohibited to talk with the other players during the experiment.

You will participate in this experiment with the other four participants. The other four participants will make up a group and play the investment game together. Each member of the group is randomly assigned a number and is named *Player[number]*. The identification number does not change during the experiment. You will not play the investment game, but participate in the experiment as the observer of the group. **The experiment consists of 10 rounds, and each round of the experiment has two stages. The group composition remains constant across periods.**

### Stage 1

In Stage 1 of each round, all four members of the group will play the investment game and each of the four players in the group is endowed with 20 tokens. Each of them has to determine the number of tokens to contribute to the project and the remaining tokens are his or hers to keep. Contributions made by all four players to the group project are multiplied by 1.6 and then distributed equally among all four players. Thus, **the gross earnings that each member makes at Stage 1 are**: **20 – (his or her contribution to the group project) + 1.6 × (contributions made by all four group members)/4**. Here are two examples:

Example 1: Each member invests 20 tokens to the project, and then each member receives 20 − 20 + 1.6 × (20 + 20 + 20 + 20)/4 = 32 tokens.

Example 2: Three members contribute all 20 tokens, one member contributes zero tokens, and then each contributing member receives 20 − 20 + 1.6 × (20 + 20 + 20)/4 = 24 tokens, and each non‐contributing member receives 20 − 0 + 1.6 × (20 + 20 + 20)/4 = 44 tokens.

### Stage 2

In Stage 2 of each round, you will participate in the experiment as the supervisor of the group. You are endowed with 32 tokens in each round. At the beginning of the stage, you will be informed about the contribution that each player has made in the investment game and then you can reduce or increase the payoff that each player receives in the investment game by assigning deduction points or increment points. Assigning 1 deduction point costs you 1 token and reduces the sanctioned player's payoff by 3 tokens; assigning 1 increment point costs you 1 token and augments the payoff of the rewarded player by 3 tokens. For example, if you assign x deduction points to a member of the group, you will lose x tokens and the member will lose 3x tokens; if you assign y increment points to a member of the group, you will lose y tokens and the member will receive 3y tokens. You have to decide how many deduction points or increment points you wish to assign to each player who has participated in the investment game. All group members will be informed of your decision. Thus, **your earnings for this round will be calculated as follows**: **32 – (the number of deduction points you assign to all four members of the group) – (the number of increment points you assign to all four members of the group)**. Then you will go to the next round. Each round of the experiment has the same stages and the earnings that you make in each round will be totaled and paid to you in cash after the experiment is over.

## 3. Decision forms and feedback cards in the TP treatment

**Round 1**—**Player1**

Your identification number is **Player1.** Please remember your identification number. Let's begin the first round. In this round, you are endowed with 20 tokens, and you have to determine the number of tokens you want to contribute to the group project, and then fill out the following decision form. The TP‐player will be informed of your decision and he or she will decide how many deduction points or increment points he or she wishes to assign to you. We will convey the feedback information to you.

**Table:**

**Contribute to the group project in this round**	
Results of **Round 1**	Player1

These are the results of Round 1, please read these results carefully. After knowing the results of this round, we will go to the next round.

Your earnings for this round

**Table:**

Your income at Stage 1
Your income at Stage 2
**Your final earnings for this round**

Round 1 information

**Table:**

Group members	Contribute to the group project	The deduction points the member receives	The increment points the member receives
You			
Player2			
Player3			
Player4			

**Round 1 Assigning deduction points and increment points to the members of the group**

Let's begin the first round. You will participate in this game as the supervisor of the group. Here are the results of Stage 1 in this round, please read these results carefully. In this round, you are endowed with 32 tokens, and you have to decide how many deduction points or increment points you wish to assign to each player who has participated in the investment game. Please complete the last two columns of the decision form, and each member of the group will be informed of your decision

**Table:**

Group members	Contribute to the group project	Deduction points	Increment points
Player1			
Player2			
Player3			
Player4			
